# Epidemiology of Shoulder Dislocations in the United States From 1990 to 2019: A Temporal Study Using the Global Burden of Disease Database

**DOI:** 10.7759/cureus.91146

**Published:** 2025-08-27

**Authors:** Gabrielle Dykhouse, Ambrose Loc T Ngo, Phillip C McKegg, Peter B Spencer, Arsalaan Sayyed, Taylor J Manes, Morgan Turnow, Nathaniel Long

**Affiliations:** 1 Medicine, Cornell University, Ithaca, USA; 2 Osteopathic Medicine, Kansas City University, Joplin, USA; 3 Orthopedic Surgery, Henry Ford Hospital, Detroit, USA; 4 Orthopedic Surgery, Ohio University Heritage College of Osteopathic Medicine, Cleveland, USA; 5 Osteopathic Medicine, Campbell University, Lillington, USA; 6 Orthopedic Surgery, OhioHealth Doctors Hospital, Columbus, USA; 7 Orthopedics, OhioHealth Doctors Hospital, Columbus, USA

**Keywords:** epidemiology, global burden of disease, incidence, prevalence, shoulder dislocation, trauma

## Abstract

Introduction

The shoulder joint is a common site for joint dislocation, with many individuals suffering from recurrent dislocations following treatment. The purpose of this study was to evaluate the epidemiology of shoulder dislocations in the United States from 1990 to 2019.

Methods

The Global Burden of Disease database was utilized to collect epidemiological data on shoulder dislocations in the United States (U.S.) from 1990 to 2019. These data included age-standardized rates of years lived with disability (YLDs), prevalence rates, and incidence rates per 100,000 people. Using the U.S. Census Bureau definitions, the data were stratified into four regions: the Northeast, Midwest, South, and West. Bartlett’s test was used to assess whether the variance of the dataset was equal. Welch’s ANOVA was performed to assess differences in YLDs, prevalence rates, and incidence rates between regions.

Results

From 1990 to 2019, there was an 8.69% decrease in mean YLDs, an 8.69% decrease in prevalence rates, and a 9.14% decrease in mean incidence rates of shoulder dislocations. Women experienced a 0.78% increase in mean YLDs, a 0.77% increase in mean prevalence rates, and a 0.27% increase in mean incidence rates of shoulder dislocation. Men experienced a 15.45% decrease in mean YLDs, a 15.45% decrease in mean prevalence rates, and a 15.82% decrease in mean incidence rate of shoulder dislocations. Regardless of region, men were more likely to experience a higher mean rate of YLDs (1.06 vs. 0.79, p<0.001), higher mean prevalence rates (17.16 vs. 12.70, p<0.001), and higher mean incidence rates (115.25 vs. 84.59, p<0.001) of shoulder dislocations. The West region experienced the highest mean rate of YLDs, the highest mean prevalence rates, and the highest mean incidence rates of shoulder dislocation. The Northeast region experienced the lowest mean rates of YLDs, mean prevalence rates, and mean incidence rates. Men experienced higher mean rates of YLDs, prevalence, and incidence of shoulder dislocations compared to women (p<0.001).

Conclusion

From 1990 to 2019, the U.S. witnessed a decline in mean YLDs, incidence, and prevalence rates for shoulder dislocations. This trend varied by gender, with men experiencing notable decreases across these metrics, while women saw slight increases. Overall, men consistently had higher rates of shoulder dislocations compared to women. Geographically, the Western region had the highest rates, whereas the Northeast had the lowest.

## Introduction

The anatomy of the glenohumeral joint allows for significant mobility at the expense of stability, placing the joint at an increased risk for dislocation [1]. The bony anatomy causes this intrinsic instability, but under normal conditions, the joint is supported by soft-tissue structures, including the glenoid labrum, glenohumeral ligaments, rotator cuff tendons, and other shoulder musculature. Trauma and chronic overuse can damage the soft-tissue stabilizers and lead to instability and frank dislocation. Dislocation most commonly occurs anteriorly, including several recognizable patterns of injury like Hill-Sachs lesions, bony and soft-tissue Bankart lesions, Bankart variants, and glenohumeral ligament avulsions [2]. From the intrinsic glenohumeral joint instability and epidemiological literature findings, there is consensus that the shoulder is the most commonly dislocated joint in the body. The incidence rate of shoulder dislocation was reported to be 8.2-26.2/100,000 person-years in multiple studies [3]. About 52.5% of shoulder dislocations are sports-related, with basketball (16.4%), American football (15.6%), and cycling (9%) being the most common sports [2].

Shoulder dislocation is reported to be influenced by sex and age. About 72.1% of all shoulder dislocations occur in men; however, the sex discrepancies vary between sports-related (86.1% men) and non-sports-related (56.7% men) dislocations. Sports-related dislocations are much more common in individuals under 21 years compared to those over 39 years (44.6% vs. 14.9%). This age-related trend is flipped when compared in non-sports-related shoulder dislocations (12% in <21 years vs. 51.7% in >39 years). For individuals over 61 years old, shoulder dislocations are more common in women. Furthermore, the age distribution for shoulder dislocation incidence is bimodal, peaking in young patients and old patients [2]. Early complications may include rotator cuff injury, fractures (e.g., Hill-Sachs lesions, greater tuberosity fractures), and neurovascular injuries to the axillary artery, axillary nerve, or brachial plexus. Later complications include recurrent instability, which often occurs due to soft-tissue damage caused by the initial dislocation event [4].

The initial treatment of patients presenting with shoulder dislocations is closed reduction. There are multiple techniques that can be used for reduction, generally classified as traction, leverage, scapular manipulation, or combination techniques. Post-reduction management usually includes immobilization unless there is another injury to the affected shoulder that may require surgical treatment. Surgical options for instability include procedures that can accomplish different goals such as limiting external rotation by tightening anterior structures, bone blocks to prevent anterior humeral head translation, osteotomies to change rotational alignment, and anatomic reconstruction of the anteroinferior capsulolabral complex. Surgery for joint instability is generally indicated for recurrent instability, pain, or functional limitation after a trial of nonoperative management [4]. There is currently a paucity of data in the literature regarding the epidemiology of shoulder dislocations in the United States. Thus, the aim of this study is to illustrate trends in the incidence, prevalence, and years lived with disability (YLDs) on shoulder dislocations in the United States from 1990 to 2019.

## Materials and methods

This study utilized the Global Burden of Disease (GBD) dataset, developed by the Institute for Health Metrics and Evaluation [5]. The GBD dataset comprises epidemiological data on 369 diseases and injuries across 204 countries from 1990 to 2019, drawing from a wide range of sources, including administrative data, census data, demographic surveys, geospatial data, and modeled data to provide estimations and projections [5]. The methods and development of the GBD dataset have been extensively documented, and its disease burden estimates have been validated [6-8]. The data were stratified into four regions: Northeast, Midwest, South, and West, according to the U.S. Census Bureau definitions [9].

Outcomes

The primary outcomes of interest included years lived with disability (YLDs), incidence, and prevalence of shoulder dislocations. YLD was selected as the primary outcome because it reflects the non-fatal burden of disease by quantifying one full year of healthy life lost due to disability or ill-health. This is particularly relevant for shoulder dislocations, which are rarely fatal but often lead to prolonged functional limitations, recurrent instability, and reduced quality of life. As such, YLD offers a more comprehensive measure of disease impact beyond incidence or prevalence alone. According to the World Health Organization, a YLD represents "one full year of healthy life lost due to disability or ill-health" [7]. Age-standardized rates of YLDs, prevalence, and incidence per 100,000 people were collected for both men and women, for each state, and the entire U.S. population. Institutional review board approval was not required as the study used de-identified, publicly accessible data.

Data extraction

We obtained data on shoulder dislocations in the United States from the GBD 2019 database using the Global Health Data Exchange (GHDx) query tool. We extracted incidence, prevalence, and YLD estimates for all ages, both sexes, and age-standardized rates per 100,000 population for each year from 1990 to 2019. Data were stratified by sex and U.S. region according to GBD definitions. All analyses were performed on these final verified datasets.

Statistical analysis

The statistical analysis methods applied in this study have been previously described and published [10]. An analysis of variance (ANOVA) was conducted to evaluate the need for multiple corrections across all measures. Bartlett’s test assessed the dataset's variance to determine if it was equal or unequal. For datasets with unequal variance, a Welch’s ANOVA was performed to examine regional differences in YLDs, prevalence, and incidence, followed by a Games-Howell post hoc test for multiple comparisons. For datasets with equal variance, Tukey’s post hoc analysis was used. Additionally, independent t-tests compared the mean YLDs, prevalence, and incidence rates between men and women by region and for the entire U.S. Statistical significance was set at p<0.05. All analyses were performed using IBM SPSS Version 29 (Armonk, NY: IBM Corp., 2022).

## Results

Data by gender

**Figure 1:**
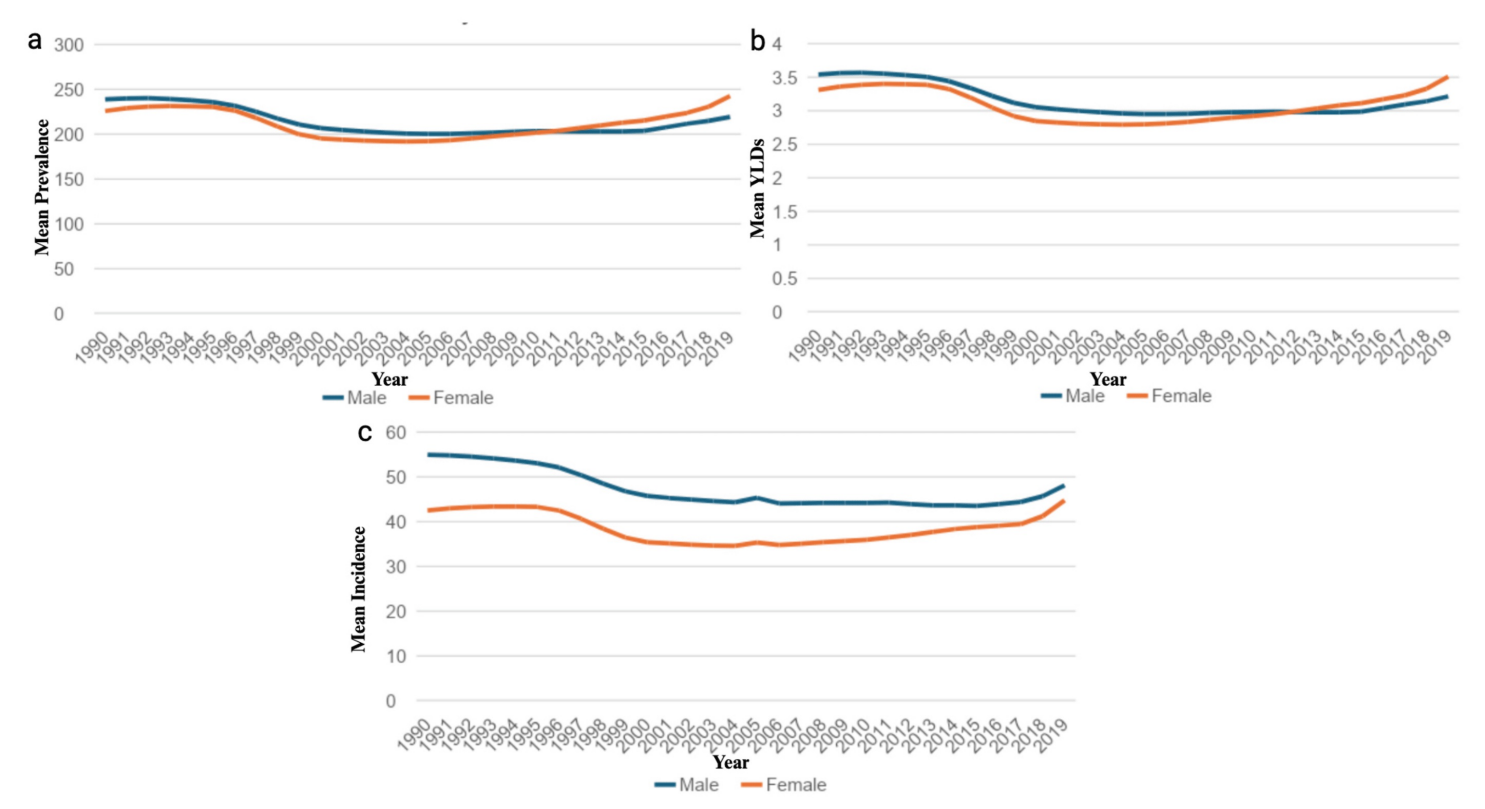
Temporal trends of years lived with disability (YLDs), prevalence, and incidence for shoulder dislocation by sex. The figures present the temporal trends from 1990 to 2019 in mean prevalence (a), mean YLDs (b), and mean incidence (c) of shoulder dislocations, stratified by sex.

From 1990 to 2019, there was an 8.69% decrease in mean YLDs, an 8.69% decrease in prevalence rates, and a 9.14% decrease in mean incidence rates of shoulder dislocations in the U.S. Women experienced a 0.78% increase in mean YLDs, a 0.77% increase in mean prevalence rates, and a 0.27% increase in mean incidence rates of shoulder dislocation. Men experienced a 15.45% decrease in mean YLDs, a 15.45% decrease in mean prevalence rates, and a 15.82% decrease in mean incidence rate of shoulder dislocations. Regardless of region, men were more likely to experience a higher mean rate of YLDs (1.06 vs. 0.79, p<0.001), higher mean prevalence rates (17.16 vs. 12.70, p<0.001), and higher mean incidence rates (115.25 vs. 84.59, p<0.001) of shoulder dislocations compared to women, which is demonstrated in Figures 1a-1c.

Data by region

**Figure 2:**
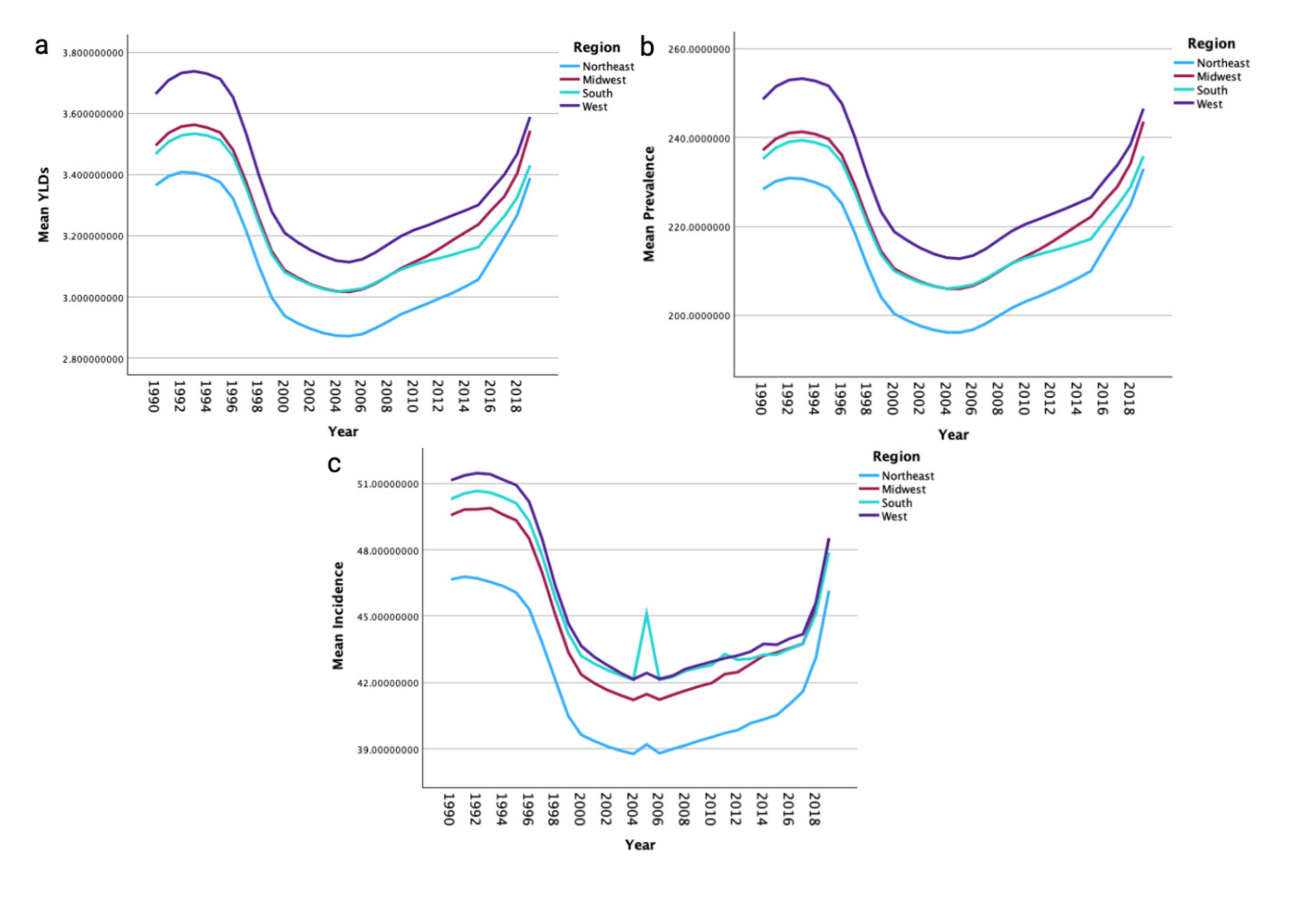
Temporal trends of years lived with disability (YLDs), prevalence, and incidence for shoulder dislocation by region. These figures illustrate temporal trends from 1990 to 2019 in mean YLDs (a), mean prevalence (b), and mean incidence (c) of shoulder dislocations, stratified by region.

The West region experienced the highest mean rate of YLDs, the highest mean prevalence rates, and the highest mean incidence rates of shoulder dislocation. The Northeast region experienced the lowest mean rates of YLDs, mean prevalence rates, and mean incidence rates (Figures 2a-2c). Men experienced higher mean rates of YLDs, prevalence, and incidence of shoulder dislocations compared to women, regardless of region (p<0.001). The South region had the highest difference in incidence between men and women (120.49 vs. 87.30).

Data by state

Table 1 displays data for each of the 50 states. North Dakota experienced the lowest decline in the mean rate of YLDs, mean prevalence rate, and mean incidence rate of shoulder dislocations. California experienced the greatest decline in the mean rate of YLDs, the mean prevalence rate, and the mean incidence rate of shoulder dislocations.

**Table 1:** Percent change from 1990 to 2019 of YLDs, prevalence, and incidence. This table displays the percent change in years lived with disability (YLDs), prevalence, and incidence of shoulder dislocations from 1990 to 2019 across different states in the United States.

State	YLDs 1990	YLDs 2019	% Change	Incidence 1990	Incidence 2019	% Change	Prevalence 1990	Prevalence 2019	% Change
Alabama	1.17	1.04	-10.50	18.83	16.85	-10.50	126.69	112.89	-10.90
Alaska	1.40	1.24	-11.71	22.58	19.93	-11.71	151.85	133.54	-12.06
Arizona	1.14	1.02	-10.17	18.37	16.50	-10.17	123.45	110.29	-10.66
Arkansas	1.24	1.13	-8.95	20.02	18.23	-8.95	134.76	122.13	-9.37
California	1.09	0.95	-13.54	17.63	15.24	-13.55	118.70	102.15	-13.94
Colorado	1.21	1.13	-7.06	19.58	18.20	-7.06	131.52	121.53	-7.59
Connecticut	1.03	0.96	-6.43	16.62	15.55	-6.44	111.71	104.03	-6.87
Delaware	1.05	0.98	-6.80	16.88	15.73	-6.80	113.39	105.33	-7.11
District of Columbia	1.05	0.98	-6.61	16.99	15.87	-6.60	114.21	106.23	-6.99
Florida	1.09	0.99	-8.88	17.53	15.97	-8.88	117.86	106.79	-9.39
Georgia	1.11	1.00	-10.06	17.86	16.07	-10.06	120.09	107.52	-10.47
Hawaii	1.04	0.98	-5.97	16.75	15.75	-5.98	112.60	105.43	-6.37
Idaho	1.16	1.05	-9.11	18.64	16.94	-9.11	125.32	113.33	-9.57
Illinois	1.11	0.98	-11.55	17.96	15.89	-11.56	120.77	106.34	-11.95
Indiana	1.07	0.99	-7.44	17.28	16.00	-7.43	116.23	107.12	-7.84
Iowa	1.09	1.04	-4.63	17.63	16.81	-4.62	118.49	112.39	-5.15
Kansas	1.09	1.03	-5.67	17.59	16.59	-5.67	118.28	110.97	-6.18
Kentucky	1.17	1.08	-7.33	18.83	17.45	-7.32	126.65	116.79	-7.79
Louisiana	1.08	0.98	-8.94	17.34	15.79	-8.94	116.71	105.82	-9.34
Maine	1.09	1.06	-2.73	17.55	17.07	-2.74	117.95	114.14	-3.23
Maryland	1.11	1.02	-8.14	17.98	16.52	-8.15	120.94	110.65	-8.51
Massachusetts	1.05	1.00	-4.75	16.99	16.18	-4.76	114.12	108.21	-5.18
Michigan	1.01	0.93	-7.27	16.26	15.08	-7.28	109.35	100.92	-7.71
Minnesota	1.16	1.09	-5.98	18.72	17.60	-5.98	125.68	117.53	-6.48
Mississippi	1.14	1.04	-8.91	18.35	16.72	-8.90	123.49	111.95	-9.35
Missouri	1.19	1.09	-8.27	19.17	17.58	-8.28	128.87	117.64	-8.72
Montana	1.17	1.11	-5.13	18.88	17.92	-5.12	126.94	119.86	-5.58
Nebraska	1.11	1.06	-4.59	17.94	17.12	-4.60	120.61	114.50	-5.07
Nevada	1.07	0.96	-10.51	17.26	15.45	-10.51	116.04	103.40	-10.90
New Hampshire	1.09	1.07	-1.78	17.63	17.31	-1.80	118.45	115.74	-2.28
New Jersey	1.17	1.04	-10.50	18.83	16.85	-10.50	126.69	112.89	-10.90
New Mexico	1.40	1.24	-11.71	22.58	19.93	-11.71	151.85	133.54	-12.06
New York	1.14	1.02	-10.17	18.37	16.50	-10.17	123.45	110.29	-10.66
North Carolina	1.24	1.13	-8.95	20.02	18.23	-8.95	134.76	122.13	-9.37
North Dakota	1.09	0.95	-13.54	17.63	15.24	-13.55	118.70	102.15	-13.94
Ohio	1.21	1.13	-7.06	19.58	18.20	-7.06	131.52	121.53	-7.59
Oklahoma	1.03	0.96	-6.43	16.62	15.55	-6.44	111.71	104.03	-6.87
Oregon	1.05	0.98	-6.80	16.88	15.73	-6.80	113.39	105.33	-7.11
Pennsylvania	1.05	0.98	-6.61	16.99	15.87	-6.60	114.21	106.23	-6.99
Rhode Island	1.09	0.99	-8.88	17.53	15.97	-8.88	117.86	106.79	-9.39
South Carolina	1.11	1.00	-10.06	17.86	16.07	-10.06	120.09	107.52	-10.47
South Dakota	1.04	0.98	-5.97	16.75	15.75	-5.98	112.60	105.43	-6.37
Tennessee	1.16	1.05	-9.11	18.64	16.94	-9.11	125.32	113.33	-9.57
Texas	1.11	0.98	-11.55	17.96	15.89	-11.56	120.77	106.34	-11.95
Utah	1.07	0.99	-7.44	17.28	16.00	-7.43	116.23	107.12	-7.84
Vermont	1.09	1.04	-4.63	17.63	16.81	-4.62	118.49	112.39	-5.15
Virginia	1.09	1.03	-5.67	17.59	16.59	-5.67	118.28	110.97	-6.18
Washington	1.17	1.08	-7.33	18.83	17.45	-7.32	126.65	116.79	-7.79
West Virginia	1.08	0.98	-8.94	17.34	15.79	-8.94	116.71	105.82	-9.34
Wisconsin	1.09	1.06	-2.73	17.55	17.07	-2.74	117.95	114.14	-3.23
Wyoming	1.11	1.02	-8.14	17.98	16.52	-8.15	120.94	110.65	-8.51

## Discussion

Between 1990 and 2019, there was a decrease in mean YLDs by 8.69%, an 8.69% decrease in prevalence rates, and a 9.14% decrease in mean incidence rates of shoulder dislocations. As a result, this suggests that there has been an improvement in the management techniques and prevention of shoulder dislocations. The reduction in incidence rate can likely be attributed to improved prehospital management of these injuries, in addition to improvements in surgical techniques for patients with glenohumeral joint instability, which would also result in a decrease in recurrence [11].

We were able to find a sex difference in which the mean YLDs, prevalence, and mean incidence rate of shoulder dislocations increased in women when compared to men across the study period. This finding is not previously reported in the literature and may be attributed to a focus on prevention in men, overshadowing women pertaining to shoulder dislocation injuries. While men have a higher incidence rate of shoulder dislocations, older women are especially vulnerable to severe traumatic shoulder dislocations. Women experience a consistent incidence of dislocation throughout their lifetime [12]. Past the age of 63, their incidence rate of shoulder dislocations surpasses that observed in men [13]. On the other hand, men were found to have decreasing mean YLDs, prevalence, and mean incidence, which may be the result of more education regarding the management of shoulder dislocations for medical personnel involved in the sports field [11]. This may be especially true considering that shoulder dislocations pertaining to men are usually due to participation in sporting and recreation activities [13]. Despite this decrease, there remains a higher mean rate of YLDs, prevalence, and incidence for men compared to women. This gender difference highlights that shoulder dislocation in men may hypothetically be the result of younger men participating in more contact-related sports compared to their female counterparts [11]. Previous authors suggest sports such as lacrosse and field hockey have contributed to the higher incidence seen in men [11].

The regional analysis showed that the mean rates of YLDs, prevalence, and incidence were high in the West and the lowest in the Northeastern region of the U.S. This may be explained by the climate and demographics, which are associated with each respective region. The generally warmer and more favorable weather found in the East is associated with an increase in physical activity time with older adults, possibly leading to the increased incidence of shoulder dislocations in the Western U.S. [14] . In addition, the Western U.S. has a greater population of young individuals who may be more active and thus see more shoulder dislocations [15]. Specifically, our group was able to find that North Dakota, compared to California, had the lowest decline in mean rate of YLDs, mean prevalence rate, and mean incidence rate of shoulder dislocations. As North Dakota is considered a rural state, it may be plagued with issues of access to healthcare providers, which would lead to a lesser decline when compared to an urban state like California [16]. Additionally, people in rural areas may not even be able to seek out healthcare due to financial issues, as rural populations are more likely to be uninsured and have inadequate access to affordable healthcare options [16].

Limitations

A key limitation of this study is that the data is retrospective and from 1990 to 2019, which may not be reflective of recent trends over the past several years. Secondly, regional discrepancies could be demonstrative of differences in infrastructure, access, and reporting protocols rather than prevalence and incidence. Furthermore, changes in coding practices over the study period may potentially affect overall estimates.

## Conclusions

From 1990 to 2019, the U.S. witnessed a decline in mean YLDs, incidence rates, and prevalence rates for shoulder dislocations. This trend varied by gender, with men experiencing notable decreases across these metrics, while women saw slight increases. Overall, men consistently had higher rates of shoulder dislocations compared to women. Geographically, the Western region had the highest rates, whereas the Northeast had the lowest. Among states, California saw the most significant decline, while North Dakota had the smallest decrease.
